# Research Progress in Fluorescent Probes for Arsenic Species

**DOI:** 10.3390/molecules27238497

**Published:** 2022-12-02

**Authors:** Yunliang Qiu, Shuaibing Yu, Lianzhi Li

**Affiliations:** 1 Department of Criminal Science and Technology, Nanjing Forest Police College, Nanjing 210023, China; 2 School of Chemistry and Chemical Engineering, Liaocheng University, Liaocheng 252059, China

**Keywords:** arsenic, fluorescence probe, nanomaterials, organic molecules, biomolecules

## Abstract

Arsenic is a toxic non-metallic element that is widely found in nature. In addition, arsenic and arsenic compounds are included in the list of Group I carcinogens and toxic water pollutants. Therefore, rapid and efficient methods for detecting arsenic are necessary. In the past decade, a variety of small molecule fluorescent probes have been developed, which has been widely recognized for their rapidness, efficiency, convenience and sensitivity. With the development of new nanomaterials (AuNPs, CDs and QDs), organic molecules and biomolecules, the conventional detection of arsenic species based on fluorescence spectroscopy is gradually transforming from the laboratory to the portable kit. Therefore, in view of the current research status, this review introduces the research progress of both traditional and newly developed fluorescence spectrometry based on novel materials for arsenic detection, and discusses the potential of this technology in the rapid screening and field testing of water samples contaminated with arsenic. The review also discusses the problems that still exist in this field, as well as the expectations.

## 1. Introduction

Arsenic (As), a non-metallic element located in group VA and period IV of the periodic table, is widely found in nature. Its average concentration in the Earth’s crust is approximately 2~5 mg/kg, which ranks as the 20th position of the elements forming the Earth’s crust. Trace amounts of arsenic can exist in soil, water, minerals, plants and normal human tissues [1]. Arsenic presents in the forms of inorganic arsenic and organic arsenic, specifically As^3+^ and As^5+^ [2]. Arsenic is a toxic and carcinogenic element. Inorganic arsenic is more toxic than organic arsenic, and As^3+^ is around 60 times more toxic than As^5+^ [3]. Inorganic arsenic species mainly exist in the form of arsenate in water, such as H_3_AsO_4_, H_3_AsO_3_, H_2_AsO_4_^−^, H_2_AsO_3_^−^, AsO_3_^3−^ and AsO_4_^3−^ [4]. Meanwhile, arsenic compounds have been used in pesticides, herbicides and insecticides for a long time, which heavily contaminates groundwater and soil, and further threatens animals, plants and human health [5]. Long-term exposure to arsenic can cause serious harm to human body, such as skin cancer, lung cancer, cardiovascular diseases and nervous system diseases [6,7]. This is due to the fact that As^3+^ can inhibit enzymes containing the sulfhydryl (-SH) group, and As^5+^ can compete with phosphoric acid in many biochemical reactions due to the instability of the bond, which soon hydrolyzes and leads to the disappearance of high-energy bonds such as adenosine triphosphate (ATP) [8,9,10]. In 2017, the world health organization (WHO) officially classified arsenic and arsenic compounds as a Group I carcinogen. The WHO recommends that arsenic concentrations in public drinking water do not exceed 10.0 μg/L [11]. Furthermore, in 2019, China’s Ministry of Ecology and Environment added arsenic and arsenic compounds to the list of toxic and harmful water pollutants. Therefore, the detection of arsenic content is of great significance for life science, environmental chemistry, medicine, agriculture and other related fields.

With scientists’ efforts, various methods have been developed for the detection of arsenic in organisms and the environment, such as atomic absorption spectroscopy (AAS) [12], atomic emission spectrometry (AES) [13], atomic fluorescence spectrometry (AFS) [14], inductively coupled plasma mass spectrometry (ICP-MS) [15], etc. In contrast to traditional methods, some novel strategies have been developed, such as using bacterial organisms to identify arsenic levels in tap water. This method mainly relies on strains with biosensing and expression functions, such as *Escherichia coli*, *Bacillus subtilis* and *Staphylococcus aureus* [16]. Although these methods have relatively high detection sensitivity, there are still a lot of disadvantages that restrict further development, such as expensive equipment, complicated operating procedures, time-consuming sample preparation and the need for trained professionals. Due to these limitations, various electrochemical and optical strategies have been extensively explored. Among them, optical detection technology, especially fluorescence probe has become an effective strategy for rapid detection of arsenic due to its simplicity, low cost, portability, selectivity, accuracy, repeatability, rapid and real-time monitoring of biological (in vivo/in vitro) [17,18] and environmental samples [19].

This review focuses on the latest progress of these arsenic fluorescent sensors. Although some excellent reviews related to arsenic detection have been published in the past decade (e.g., electrochemical, biosensors and nanomaterial-based sensors) [20,21,22,23], there are few comprehensive reviews of fluorescence sensors for arsenic analysis. In particular, due to the widespread attention and rapid development of this filed, the latest research results of arsenic-based fluorescent chemical sensors must be summarized. Therefore, this review systematically sorts out different types of arsenic fluorescence sensors and summarizes their applicable conditions and the advantages of various methods. Finally, we present our perspective on future progress. We hope that this review will be enlightening to readers who wish to work in this field in the near future.

## 2. Nanomaterial Based Probes

Compared with the traditional detection methods, the introduction of nanomaterials with attractive optical, electrochemical and catalytic properties opens a new avenue for the detection of arsenic. In this subsection, various nano-arsenic sensors based on their structure, morphology and characteristics are compared. Table 1 lists some reported fluorescence sensors based on nanomaterials for arsenic detection.

### 2.1. Functionalized Gold Nanoparticles

Gold nanoparticles (AuNPs) are generally between 1~100 nm in size and show different colors with certain particle sizes. Therefore, AuNPs are attractive as sensing materials. Based on the characteristics of AuNPs, in 2012, Banerjee et al. reported an arsenic sensor based on dipeptide (Cys-Cys) modified water-soluble fluorescent gold clusters (AuCs) [24]. The synthesized AuCs exhibited interesting fluorescence properties, including a large Stokers shift (110 nm), a quantum yield (QY) of 41.3% and photochemical stability. Moreover, As^3+^ could be selectively detected in the presence of other metal ions in the aqueous solution, and the limit of detection (LOD) was 53.7 nM, which was much lower than the permissible limit of arsenic in drinking water (133 nM) stipulated by WHO.

In contrast to traditional functionalized AuNPs, there are some novel strategies that have already been reported. Doble et al. developed a sensitive strategy for As^3+^ detection based on the fluorescence-quenching system of bovine serum albumin (BSA) capped carbon-gold complex (C-Au-BSA) [25]. The method consisted of two steps, first synthesizing carbon-gold (C-Au) complex, and then covering C-Au with BSA to form C-Au-BSA complex. In this system, C-Au and BSA acted as energy donor and energy acceptor, respectively. The photoluminescence (PL) spectrum of C-Au-BSA varied in the presence of As^3+^. As^3+^ could bind to BSA and unbind it from C-Au composites to reduce PL efficiency, so it could be used to determine the concentration of As^3+^ with LOD as low as 0.004 ppb and had been successfully applied to the detection of As^3+^ in drinking water. Wang et al. reported a probe for As^3+^ detection by combining polyethyleneimine-coated persistent luminescent nanoparticles (PLNPs) and AuNPs modified with DTT [26]. The synthesized probe could be used for As^3+^ detection through near infrared (NIR)-emitting inner filter effect (IFE). The assay could detect concentrations ranging from 0.067 to 13.4 μM with an LOD of 55 nM.

Based on the fluorescence resonance energy transfer (FRET) between AuNPs and fluorophore, the unique optical effect of AuNPs, and the high affinity and high specificity of aptamers, Yuan et al. established a fluorometric and colorimetric dual-mode detection method for As^3+^ [41]. In this method, arsenite aptamer modified by the fluorescence probe FAM-Apt was adhered on the surface of unmodified AuNPs. The FRET occurred between FAM-Apt and AuNPs, resulting in fluorescence quenching. When existing in the system, arsenite combined with FAM-Apt, leading to the release of FAM-Apt from the surface of AuNPs and the enhancement of fluorescence. Meanwhile, AuNPs without FAM-Apt protection accumulated in salt solution, with the color changed from red to blue-gray. Therefore, As^3+^ can be detected by fluorescent and colorimetric dual modes.

### 2.2. Carbon Dots

Carbon dots (CDs), also known as carbon quantum dots (CQDs) or carbon nanodots (CNDs), are a class of zero-dimensional carbon nanomaterials with significant fluorescence properties. CDs are composed of ultra-fine, dispersed, quasi-spherical carbon nanoparticles with diameters below 10 nm. CDs have the characteristics of adjustable fluorescence, biocompatibility, photostability and chemical stability. These unique advantages make CDs potentially applicable in bioimaging, sensing, drug delivery, optoelectronic devices, catalysis, energy conversion, etc.

Nandi et al. used citric acid and sodium thiosulfate to synthesize multicolor fluorescent sulfur-doped CDs in one-step [27]. The synthesized CDs had the bifunctional capability to detect both As^3+^ and GSH by visualization. Furthermore, the newly developed CDs were very specific for As^3+^, even in the interference of high concentrations of other metal ions. In addition, the LOD of 32 pM was sufficient to meet the minimum concentration stipulated by WHO. Using edible pear and cactus fruits as carbon sources, Panneerselvam et al. passivated glutathione (GSH) on CDs surfaces to form a sensor with excellent optical properties and water solubility [28]. The simple sensing platform developed by GSH-CDs was highly sensitive and selective with an “off” fluorescence response for dual detection of As^3+^ and ClO^−^ in drinking water. The LOD of As^3+^ and ClO^−^ were 2.3 nM and 0.016 μM, respectively, which could be used for the analysis of real environmental samples. This approach had great development potential, both in terms of environmental protection and economic benefits. Karak reported an in-situ fabrication of polyaniline nanofiber/carbon dot nanomaterials with excellent antioxidant properties for fluorescence detection of As^3+^ in contaminated water with an LOD of 0.001 ppb [42]. Nayak et al. had also developed sulfur-doped CQDs for the selective detection of toxic arsenite in water [43]. Functionalized sensing for arsenite in water was demonstrated by the “on” fluorescence mechanism, which reduced the likelihood of false-positive signals associated with the previously reported “off” mode.

**Figure 1:**
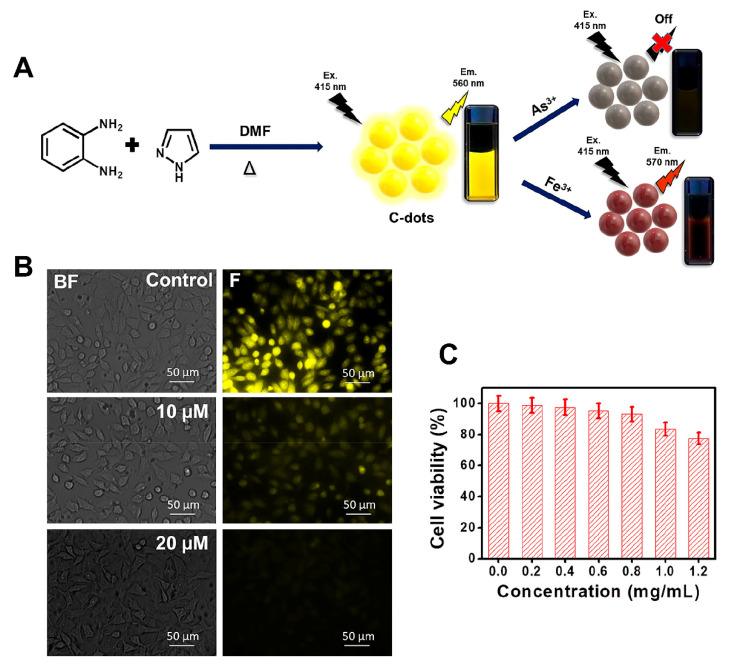
(**A**) Schematic Illustration of the synthesis process and selective ion recognition of yellow fluorescent CDs; (**B**) Fluorescence images of living cells for As^3+^ ion detection; (**C**) Determination of biocompatibility of CDs based on MTT assay [29]. Adapted with permission from Ref. [29]. Copyright 2021, American Chemical Society.

In contrast to the theoretical bases of the aforementioned methods, Bhunia et al. reported a paper strip in 2021 that could be applied to detect both As^3+^ and Fe^3+^ in field applications [29]. Bright yellow fluorescent CDs were prepared with a mixture of *o*-phenylenediamine and pyrazole (Figure 1A). Metal ion sensing studies confirmed that CDs aqueous dispersions could selectively detect As^3+^ and Fe^3+^ after strong fluorescence quenching, and the detection limits were 24.4 nM and 63.4 nM, respectively. This method had not only been successfully applied to the detection of ions in real water samples, but also successfully carried out the intracellular detection of ions in living cells with lower concentrations (Figure 1B,C).

Due to the strong surface plasmon resonance (SPR) effect of AuNPs, visual detection of As^3+^ based on AuNPs has also been investigated. Li et al. constructed a colorimetric and fluorescence dual-mode optical nanosensor for accurate detection of As^3+^ in water [44]. The nanosensor consisted of AuNPs modified with trithiocyanate (TMT-AuNPs) and amino-functionalized carbon dots (NCDs). After mixing TMT-AuNPs and NCDs, the fluorescence of NCDs was weakened due to UV overlap. In the presence of As^3+^, As^3+^ coordinates with the sulfhydryl group of TMT-AuNPs, resulting in an aggregation of TMT-AuNPs, which could be used for colorimetric detection with an LOD of 0.87 ppb. Meanwhile, the fluorescence of NCDs was recovered and could be used for fluorescence detection with an LOD as low as 0.66 ppb.

### 2.3. Quantum Dots

Quantum dots (QDs) are nanoscale semiconductors that emit light at a specific frequency by applying a certain electric field or light pressure to the material. The frequency of the light emitted varies with the size of the semiconductor, so the color of the light emitted can be controlled by adjusting the size of the nano-semiconductor. Meanwhile, QDs are characterized by good photostability, wide excitation spectrum and narrow emission spectrum, large Stokes shift, good biocompatibility and long fluorescence lifetime. Therefore, optical sensors based on QDs are particularly attractive. Paria et al. reported a silver doped hollow CdS/ZnS bi-layer (Ag-h-CdS/ZnS) nanoparticle for simple fluorescence determination of As^3+^ in the aqueous phase [30]. There was a good linear relationship between the fluorescence-quenching intensity and the concentration of As^3+^ in the range of 0.75–22.5 µg/L. The LOD was as low as 0.226 µg/L at neutral pH. In addition to the common CdS/QDs, CdSe/QDs and CdTe/QDs have also been reported. Qiu et al. developed a novel CdSe/QDs coated with a Tb^3+^ complex of guanosine monophosphate (Tb-GMP) [31]. The nanoprobe formed by the combination of carboxylated QDs, GMP with phosphate group and Tb^3+^ could exhibit two emission peaks under the excitation at 280 nm, corresponding to QDs (652 nm) and Tb-GMP (547 nm), respectively. In the absence of As^5+^, Tb-GMP was decomposed by acid phosphatase (ACP), resulting in a decrease in fluorescence intensity at 547 nm, which had no effect on the fluorescence intensity of QDs at 652 nm. In contrast, ACP activity was inhibited due to competitive binding in the presence of As^5+^, so the concentration of As^5+^ could be determined by the fluorescence intensity ratio at 547 and 652 nm, with the LOD being 0.39 ppb. Meanwhile, under the UV lamp, it could be visually observed that, with the increase of As^5+^ concentration, the probe showed a distinguishable color change from green to red.

**Figure 2:**
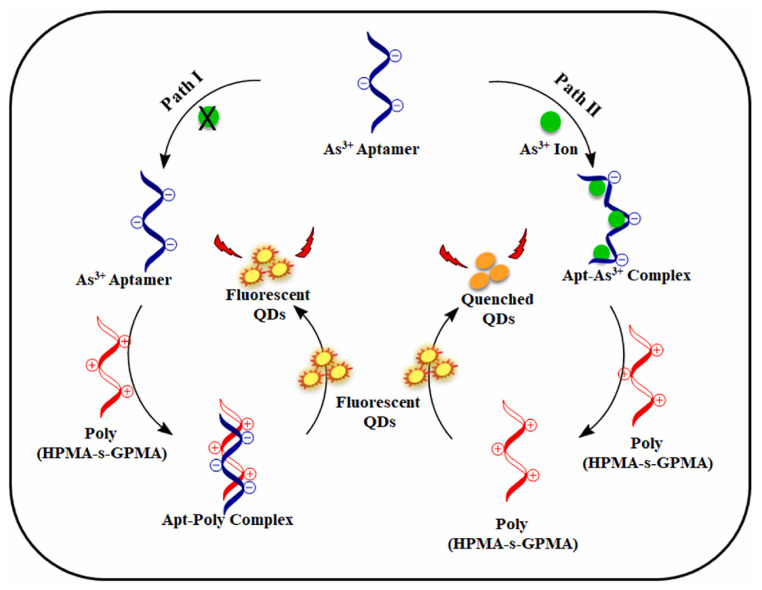
Schematic representation of As^3+^ detection mechanism [33]. Adapted with permission from Ref. [33]. Copyright 2022, Elsevier.

Similar work has been reported by Songsrirote et al., in which a simple paper-based device for detecting arsenic in water samples was designed by using thiol succinate capped CdTe/QDs (MSA-CdTe/QDs) as the detection probe [32]. The synthesized QDs were coated on the paper strip to react with the generated arsine gas (AsH_3_). In the presence of arsenic, the fluorescence emission of MSA-CdTe/QDs was quenched with a detection limit of 0.016 mg/L. Recently, Sharma et al. reported a poly HPMA-s-GPMA (HPMA: N-(2-hydroxypropyl)methacrylamide; GPMA: N-(3-guanidinopropyl)methacrylamide) copolymer for the preparation of a three-component aptasensor for the simple, selective, rapid and label-free detection of As^3+^ [33]. As shown in Figure 2, the sensor consisted of HPMA-s-GPMA copolymer, mercaptopropionic acid capped CdTe@CdS/QDs (MPA-CdTe@CdS/QDs) and As^3+^-specific aptamer. In this system, As^3+^ could bind to the specific aptamer, allowing HPMA-s-GPMA to be freely used for electrostatic interactions with QDs to quench the fluorescence signal. The LOD was 246.77 pM in the range of 0.01–100 nM. This “on–off” fluorescent aptamer sensor was highly selective to target ions and has a good application prospect. Zhang et al. used carboxyl modified red fluorescent CdTe to design colloidal QDs chains, which achieved self-assembly via the mediation of trithiocyanuric acid (TTCA) [45]. A ratiometric fluorescence sensor with dual-emission was fabricated by mixing QDs chains with blue fluorescent CDs for the visual ultra-sensitive detection of As^3+^ in ambient water. The LOD of this proportional fluorescent probe could be as low as 1 ppb.

In contrast to the common Cd-based QDs, some novel strategies of QDs have also been reported. Zinc oxide (ZnO) QDs without stabilizers were synthesized by refluxes of zinc acetate dihydrate in methanol under alkaline conditions, which could selectively detect As^3+^ and As^5+^ by fluorescence-quenching response [46]. The detection limits of As^3+^, As^5+^ and As^3+^+As^5+^ were 27, 7 and 28 ppb, respectively. Meanwhile, ZnO/QDs had a good anti-interference ability, which could be applied to practical sample detection. Similarly, Halder et al. have developed a thiosalicylic acid-capped ZnS/QDs fluorescence sensor which can simultaneously detect As^3+^ and As^5+^ through the photoluminescence “turn-on” characteristic [47]. Anbia et al. designed an L-methionine (Meth) capped PbS/QDs with uniform size, intensive fluorescence emission (510 nm), 17.3% QY, intensive photo-stability and robust time-stability [34]. In the presence of As^3+^, the fluorescence intensity was effectively quenched due to the strong interaction between As^3+^ and Meth to form the S, N bidentate chelate ring. A linear relationship between fluorescence intensity and As^3+^ concentration was observed in the range of 5 to 150 ppb, with an LOD of 3.7 ppb. A novel oligonucleotide functionalized CuInS_2_ QDs@magnetic Fe_3_O_4_ nanocomposite fluorescent “off–on” nanosensor was applied to arsenate detection [35]. Amino-terminal oligonucleotides were first covalently linked to carboxyl groups on the surface of CuInS_2_ QDs to form oligonucleotide-functionalized CuInS_2_ QD (ssDNA-CuInS_2_ QDs). The Fe_3_O_4_ nanoparticles then adsorbed to the ssDNA-CuInS_2_ QDs, resulting in fluorescence quenching. Due to competitive binding, arsenate bound to Fe_3_O_4_ first, leading to the turn on of fluorescence, and the LOD was 0.13 nM. Similarly, Qiu et al. also reported a novel fluorescent G-/T-rich ssDNA-QDs, which was synthesized by hydrothermal treatment at a relatively low reaction temperature [36]. The obtained ssDNA-QDs possessed unique optical properties and maintained the basic structural and biological activities of ssDNA precursors, which enabled ssDNA-QDs to bind specifically to arsenite and form ordered assemblies, and lead to an increase in fluorescence intensity. A linear relationship was measured between 1 ppb to 150 ppb, with an LOD of 0.2 ppb. The QDs sensor showed excellent selectivity for arsenite determination and had good anti-interference ability, which was convenient for its application in complex practical water analysis. Bose et al. has fabricated a highly sensitive and selective magnetic graphene quantum dots (Fe-GQDs) sensor for fluorescent “turn-on” mode detection of As^3+^ [37]. After the addition of As^3+^ to Fe-GQDs, the aggregation-induced emission (AIE) occurred due to the restriction of intramolecular vibration motion, and the Fe-GQDs aggregates were formed. The resultant LOD value for Fe-GQDs was 5.1 ppb, which was well below the permissible limit of arsenic in drinking water.

Dipsticks for detecting arsenic based on QDs have also been reported. Han reported a fluorescent colorimetric strip with a very broad/continuous “red to cyan” response to the presence of As^3+^ [48]. QDs were modified with GSH and DTT to obtain the super-sensitivity to As^3+^ by the quenching of red fluorescence through the formation of dispersive QDs aggregates. A small number of cyan CDs with spectral blue-green components as the photostable internal standard were mixed into the QDs solution to produce a composited red fluorescence. After adding As^3+^ to the solution, the fluorescence color could gradually change from red to cyan with an LOD of 1.7 ppb. When the sensing solution was printed on a sheet of filter paper, the addition of As^3+^ showed a range of color changes and clearly resolved the dosage scale as low as 5 ppb. This study provided a convenient, fast and low-cost method for on-site As^3+^ detection.

### 2.4. Other Types of Nanosensors

Arsenic contamination has adverse public health effects and seriously affects millions of people worldwide. Therefore, sensitive and selective monitoring of arsenic in water is highly attractive and challenging. Qiu et al. proposed novel luminescent Ce^3+^-based coordination polymer nanoparticles (Ce-CPNs) for the selective detection of As^3+^ [38]. The synthesized Ce-CPNs were dispersed and exhibited a fluorescence peak at 353 nm when excited at 280 nm. The presence of As^3+^ could induce the aggregation and subsequent π-π stacking of Ce-CPNs, which led to fluorescence quenching. The LOD this method for As^3+^ could be as low as 0.7 ppb under optimal conditions. Sivanesan developed an As^3+^ fluorescent biosensor based on MoS_2_ nanosheets [39]. FAM-labeled arsenic aptamer (Ars-3) ssDNA was used as the signal reporter, and MoS_2_ nanosheet was used as the quencher. In the presence of As^3+^, FAM-labeled Ars-3 ssDNA could bind to As^3+^ through phosphate and amine groups to form a G-quadruplex structure, resulting in an increase in fluorescence intensity with an LOD of 18 nM. Zhang et al. reported a bimetallic Cd/Zr-UiO-66 material for fluorescence sensing of trace amounts of As^5+^ and Fe^3+^ [40]. Interestingly, the obtained bimetallic Cd/Zr-UiO-66 could be used as an “on” probe for As^5+^ and an “off” probe for Fe^3+^ with LODs of 5.4 μM and 4.3 μM, respectively.

An Eu:Y_2_O_3_ nanophosphor (EYN) and EYN dispersed polyvinyl alcohol (PVA) fluorescent film (EYF) were synthesized by Dwivedi and used for the detection of As^3+^ [49]. The fluorescence intensity of EYF decreased rapidly with the increase of As^3+^, which might be due to the reduction in the population of Eu^3+^ in the ^5^D_0_ level and the formation of direct or indirect coordinate bonds with Eu^3+^. The fluorescence intensity of the EYF nanoprobe was completely quenched at 260 µg/L concentration of As^3+^, and the LOD was 57.5 ng/L (=0.057 ppb) in the linear range of 0–100 µg/L. Kumar et al. developed a sensitive α-NaYF_4_:Yb^3+^, Er^3+^ conversion platform using Moringa oleifera leaf extracts for the detection of As^3+^ in drinking water [50]. The presence of polyphenols in leaf extracts induced luminescence resonance transfer (LRET), which reduced Er^3+^ emission (red and green band) upon excitation at 980 nm. This method could detect As^3+^ in drinking water at concentrations below 10 ppt. Xu et al. also prepared a multifunctional Fe_3_O_4_@NaGdF_4_:Yb:Er (Fe@UCNPs) based on LRET with both fluorescence and magnetic properties based on LRET for detecting the As^3+^ [51]. Arsenic aptamer-modified dual-functional magnetic upconversion nanoparticles and cDNA-modified tetramylrhodamine (TAMRA) fluorophore constitute an energy donor–acceptor pair. The hybridization of aptamers and cDNA reduced the distance between UCNPs and TAMRA and induced the occurrence of LRET, which effectively quenched fluorescence. However, arsenic could specifically bind to aptamers and interfered with the LRET process, leading to the recovery of UCNPs fluorescence. The probe could amplify the detection signal, significantly shorten the detection time and improve the detection sensitivity.

## 3. Organic Molecule-Based Probes

### 3.1. Small Organic Molecules

In recent years, some fluorescent probes based on organic small molecules have been reported [10,52]. In this regard, the fluorescence detection technique based on organic small molecules has achieved an unprecedented success, not only because of its low detection limit, but also due to its low cost, easy operability and high selectivity [53]. In fact, fluorescence probes are widely used to detect biologically relevant analytes.

**Figure 3:**
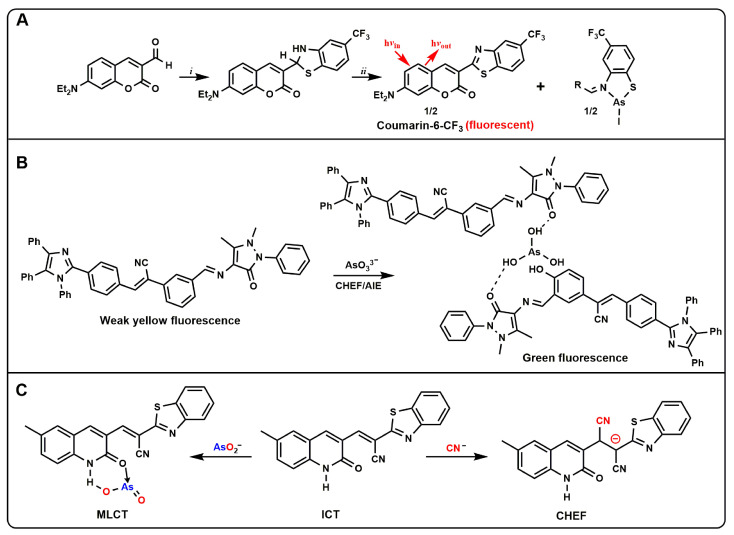
(**A**) Synthesis of AF1 and proposed As^3+^ response; (**B**) Schematic of As^3+^ detection using the probe TBAB; (**C**) The Proposed mechanism of chromogenic and fluorescence enhancement of the probe in the presence of AsO_2_^−^ and CN^−^.

Harrop et al. developed a small-molecule probe ArsenoFluor1 (AF1, Figure 3A) [54]. The AF1 probe could be combined with As^3+^ and produce a highly fluorescent benzothiazole molecule, a common dye called coumarin-6. The LOD was estimated to be 0.53 nM (0.24 ppb), suggesting that AF1 might be used to monitor As^3+^ levels well below the EPA standard of 10 ppb. Based on this structure, the questionable set also replaced -CF_3_ with -H, which could also be applied to the detection of As^3+^, and expanded the database of AF1 probes [55]. Song et al. reported a new tetraphenylimidazol-based probe (TBAB, Figure 3B) functionalized with Schiff base for the detection of arsenic ions in water [56]. After the addition of arsenic ions, the chelation of TBAB with arsenic activated the AIE properties, resulting in enhanced fluorescence and a distinct fluorescence change from pale yellow to green that was visible to the naked eye. The probe could selectively detect arsenic in the presence of interfering substances with an LOD less than 0.7 ppb, which was well below the limit set by the WHO. A pyrene-based oxacalix [4]arene-Ce^3+^ complex (L–Ce) based on light-induced electron transfer (PET) and chelation-enhanced fluorescence (CHEF) was developed [57]. The complex could selectively detect As^5+^ and Cr^6+^ through a fluorescence-quenching response simultaneously. This L–Ce “turn-off” fluorescent probe for the detection of AsO_4_^3−^ and CrO_4_^2−^ could achieve an LOD of 2 ppb and 93 ppb, respectively. Similarly, Kumar et al. reported a quinoline acrylonitrile probe based on metal–ligand charge transfer (MLCT) and CHEF (Figure 3C) [58]. The probe was prepared from 6-methyl-2-oxo-1,2-dihydroquinoline-3-carbaldehyde with benzothiazole-2-acetonitrile. The probe showed a selective “off–on” fluorescence response to AsO_2_^−^ and CN^−^, and could simultaneously detect arsenate by colorimetric visualization. The probe could bind to AsO_2_^−^ and CN^−^ in a 1:1 ratio and was undisturbed by other competing ions in the pH ranges 2–10 and 3–8, respectively. The LOD of AsO_2_^−^ by using spectrophotometry and RGB color tool were measured to be 24 ppb and 498 ppb, respectively, while CN^−^ detection by spectrofluorimetry detected down to 1 ppb. The probe was successfully applied to the detection of cyanide and arsenite in tap water and well water.

2,4-dinitrophenyl hydrazones are of great interest because they can interact with analytes via forming strong hydrogen bonds. Padmini et al. developed a visual probe based on 2,4-dinitrophenyl hydrazine framework for the detection of highly toxic As^3+^ in water [59]. Probe PHTH (E)-4- [{2-(2,4–dinitrophenyl) hydrazono}benzene 1,3-diol] was synthesized by 2,4-dinitrophenyl hydrazine, 2,4-dihydroxy benzaldehyde and ethanol. Experimental studies clearly demonstrated the high selectivity of the probe for As^3+^ compared with other competing metal ions. Furthermore, the addition of As^3+^ to the probe caused the PHTH solution to change from orange to purple under UV light in DMSO solvent. The LOD of the aqueous medium was calculated to be 0.35 μM.

**Figure 4:**
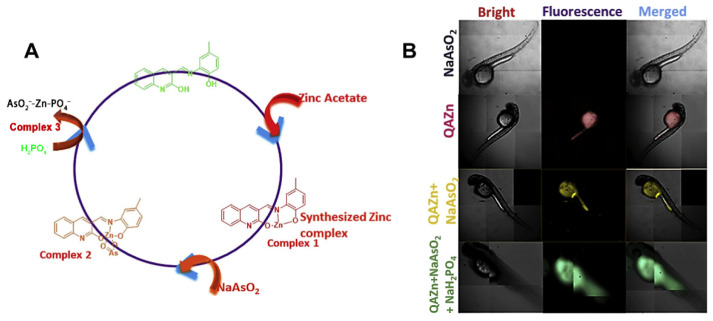
(**A**) Possible mechanism of the chemosensor QAZn with AsO_2_^−^ and H_2_PO_4_^−^; (**B**) HCS microscopy of the cell imaging of NaAsO_2_, QAZn, QAZn + NaAsO_2_ and QAZn + NaAsO_2_ + NaH_2_PO_4_ [60]. Reproduced with permission from Ref. [60]. Copyright 2019 Elsevier.

Arsenic detection has not only been widely used in ambient water samples, but also in the development of intracellular detection. Annaraj et al. reported a fluorescent probe for the simultaneous detection of AsO_2_^−^ and H_2_PO_4_^−^ in zebrafish embryos [60]. The probe was based on the red fluorescent zinc complex (QAZn) and had an extremely strong direct binding to AsO_2_^−^ and H_2_PO_4_^−^ (Figure 4A). In the presence of AsO_2_^−^, it was firstly bound to the QAZn and triggered the intramolecular charge transfer (ICT) process, which led to a blue shift of the emission peak. After that, the addition of H_2_PO_4_^−^ caused the dissociation of Zn(II) from QAZn-AsO_2_ complex and the released free ligand restored green fluorescence. Meanwhile, it has been further exploited for live cell studies in zebrafish embryos (Figure 4B). Rhodamine, as a kind of dye with excellent photochemical properties, is often used as a fluorescent signal molecule. Banerjee et al. designed a molecular probe rich in rhodamine, PBCMERI-23 (3′,6′-bis-(ethylamino)-2-((2-hydroxy-5-methylbenzylidene)amino)-2′,7′-dimethylspiro [isoindoline-1,9′-xanthen]-3-one) [61]. The simple, immediate and cost-effective luminescence probe enabled As^3+^ to be selectively detected in aqueous media at an LOD level of 0.164 ppb. Meanwhile, the level of As^3+^ was monitored in different cells, and the probe showed flashing yellow fluorescence, which indicated that the probe had good cell permeability and biological applicability. Recently, this group has synthesized a naphthalene additive luminophore *N*-((4-((naphthalene-5-ylimino) methyl) phenyl) methylene) naphthalene-1-amine (NPN) with various properties. Based on “arsenoselective azomethine hydrolysis” (ASAH), NPN exhibited excellent fluorescence performance when interacting with As^3+^. NPN could monitor As^3+^ in different natural water sources in real time by a “turn on” fluorescence response. Meanwhile, cells treated with As^3+^ emitted a flashing blue emission fluorescence. The rapid fluorescence enhancement capability of the probe enhanced its potential for field applications.

### 3.2. Organic Frameworks

Metal organic frameworks (MOFs) are a class of crystalline porous material with a periodic network structure. They are composed of inorganic metal centers (metal ions or metal clusters) and bridged organic ligands connected to each other through self-assembly. Since the emergence, MOFs have shown great potential in a wide range of applications [62]. In fact, MOFs exhibit excellent luminescence properties due to their π-rich bridging organic ligands as well as metallic nodes/clusters and adsorbed or functionalized luminescent guest molecules. In recent years, luminescent MOFs (L-MOFs) have exhibited potential in a variety of applications including environmental problems [63,64,65,66].

**Figure 5:**
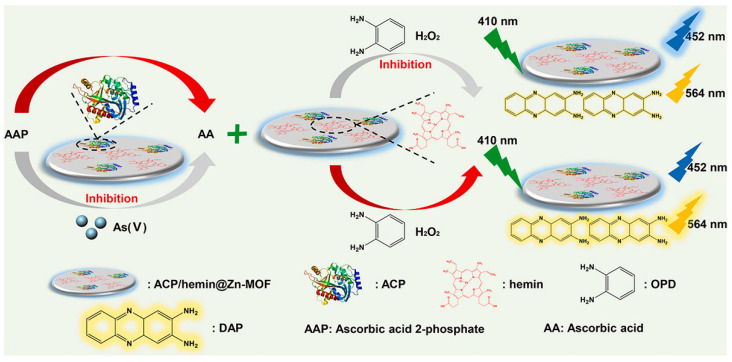
Description of ratio fluorescence detection of As(V) using ACP/hemin@Zn-MOF as a multifunctional sensor [68]. Reproduced with permission from Ref. [68]. Copyright 2022 Elsevier.

Ghosh et al. synthesized a new hydrolytically stable luminescent Zn^2+^ based cationic MOFs (iMOF-4C) [67]. iMOF-4C was constructed from a tripodal neutral N-donor linker 1,1′-(5′-(4-(1*H*-imidazol-1-yl) phenyl)-[1,1′:3′,1″-terphenyl]-4,4″-diyl)bis(1*H*-imidazole) and a d^10^ metal ion for the simultaneous detection and removal of environmentally toxic oxygen anions (CrO_4_^2−^, Cr_2_O_7_^2−^, HAsO_4_^2−^, and HAsO_3_^2−^) in aqueous media. The MOF based sensing probe showed accurate and sensitive recognition of these anions through its unique “on–off” fluorescent signal output and extremely fast response time. Xu et al. developed a ratiometric fluorescent biosensor based on acid phosphatase and hemin loaded multifunctional Zn-based MOF (ACP/hemin@Zn-MOF) for As^5+^ sensing [68]. As shown in Figure 5, the intrinsic fluorescence (452 nm) in ACP/hemin@Zn-MOF was derived from 2-aminoterephthalic acid ligand, and the hemin was characterized by peroxidase activity. ACP/hemin@Zn-MOF could catalyze the oxidation of o-phenylenediamine (OPD) to produce 2, 3-diaminophenazine (DAP) with a fluorescence signal at 564 nm and weaken the fluorescence intensity of ACP/hemin@Zn-MOF (452 nm). When present in the sample, ascorbic acid 2-phosphate (AAP) could be hydrolyzed by ACP to form ascorbic acid (AA), which hindered the oxidation of OPD and thus affected the fluorescence intensity at 564 nm. Furthermore, the addition of As^5+^ could irreversibly poison ACP to prevent hydrolysis of AAP, resulting in the recovery of the fluorescence signal at 564 nm and the suppression of the signal at 452 nm again. The LOD of ACP/hemin@Zn-MOF for As^5+^ was 1.05 μg/L. Based on the optimization of organic ligands, a novel Eu-MOF with the capability of AsO_4_^3−^ emission sensing and trapping was constructed by the solvothermal method [69]. The Eu-MOF nanostructures with dispersion characteristics clearly showed a “turn-on” fluorescence emission characteristic with obvious intensity contrast in the arsenate sample, which reduced the LOD to 17.8 nM for arsenate species in the aqueous environment. Subramanian et al. reported an MOF-derived magnetic porous carbon (MPC) composite [70]. FAM labeled ssDNA was adsorbed on the surface of MPC composites and fixed by π-π stacking interaction, which resulted in the quenching of fluorescence intensity. When As(V) was added to the probe system, the strong binding of As(V) to MPC resulted in the spontaneous displacement of FAM-labeled ssDNA from the MPC material, and thus the fluorescence intensity was restored. Based on this principle, the fabricated sensor exhibited a highly sensitive fluorescence response to As^5+^ in the range of 0–15 nM, with an LOD as low as 630 pM.

**Figure 6:**
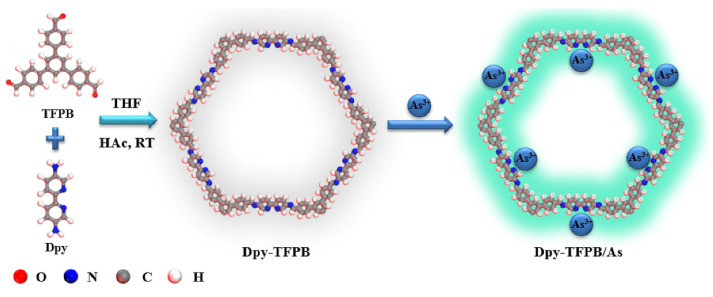
A diagram summarizing the synthesis of Dpy-TFPB [72]. Reproduced with permission from Ref. [72]. Copyright 2021 Elsevier.

In recent years, covalent organic frameworks (COFs) have attracted much attention in fluorescence sensing due to their regular pore structure, stable π-conjugate frame, and easiness of synthesis [71]. COFs are easy to functionalize and uses the specified group as the recognition sites in the framework. In particular, 2D COFs may be the best candidate for the detection of highly toxic ions such as As^3+^. Yin and Liu reported functional COFs based on bipyridine (Dpy-TFPB) for the fluorescence “turn on” mode detection of As^3+^ [72]. The synthesis method and structure of Dpy-TFPB are shown in Figure 6. The nitrogen-based site of Dpy-TFPB was a highly selective receptor for As^3+^, and its π bond acted as a signal responder. In the presence of As^3+^, the combination with N group destroyed the photoinduced electron transfer (PET) process and exhibited obvious fluorescence. Dpy-TFPB showed high sensitivity and an ultra-low LOD of 8.86 nM was determined. To further improve the application of COFs in the detection of arsenic, Chen et al. used COFs as a fluorescence sensor for the first time to detect and adsorb organic arsenic in water [73]. Two isoreticular crystalline and highly porous sp^2^ carbon-conjugated COFs were synthesized, and were amidoxime-functionalized via post-synthetic modification (PSM). The long-range ordered and π-conjugated system ensured that two kinds of COFs were used as fluorescence sensors for the detection of representative organic arsenic roxasone (ROX) through a fluorescence-quenching response. The LOD of ROX for the two kinds of COFs were 6.5 nM and 12.3 nM, respectively.

## 4. Biomolecule and Cell-Based Probes

There is growing awareness of the potentially harmful effects of anthropogenic pollution on human and environmental health. Excess arsenic not only contaminates water and soil, but ultimately affects the health of living organisms. Despite the high risk of heavy metals, the detailed mechanisms underlying their overall biotoxicity have not been fully elucidated. Therefore, there is an urgent need for a convenient, real-time, and low-cost method to detect arsenic and evaluate relative bioavailability. The establishment of biomolecule-based methods, especially the use of proteins, microorganisms, cells and other biosensors to detect and quantify arsenic content has become an important topic in current scientific research [74,75].

### 4.1. Peptide/Proteins

Peptides are formed by the dehydration and condensation of amino acids, which can be used as polydentate ligands and can rapidly bond with metal ions to form very stable chelates. Therefore, it has high sensitivity for metal ion detection. As early as 2012, Ahmad et al. had studied the application of dansylated peptide in As^3+^ detection. A probe with peptide sequence Dansyl-*D*-Ala-Gly-OH (DAG) was designed [76]. The addition of As^3+^ could combine with N and O atoms in DAG to form a 1:1 complex, resulting in fluorescence quenching to achieve the purpose of As^3+^ detection. The LOD of DAG for As^3+^ was 0.15 μM. The developed detection method had good anti-interference abilities and has been applied to the analysis of electroplating wastewater samples. Compared with other metal ion probes based on polypeptides, there is less research on arsenic detection. The multiple binding sites and biocompatibility of peptides enable them to show application prospects in the detection of arsenic, and researchers are encouraged to study this field further.

Metal ions play a key role in many life processes and exist in many proteins, such as hemoglobin, myoglobin, hemocyanin and zinc finger protein, etc. Therefore, it is an important content of life science to study the binding of metal ions and proteins. Turner et al. designed a probe for the first time by using different classes of transcriptional regulatory proteins genetically and chemically modified to detect arsenic, hydroxylated polychlorinated biphenyls (OH-PCBs) and cyclic AMP (cAMP) [77]. In recent years, both synthetic probes and genetically encoded biosensors have been extensively developed. For example, unlike exotic synthetic dyes, genetically encoded sensors (fluorescent protein (FP)-based biosensors) have low perturbations to intracellular metal homeostasis [78]. By fusing with specific signal sequences, genetically encoded sensors can be selectively localized to suborganelles. Yun et al. reported a genetically encoded As^3+^ sensor [79]. They introduced the mutation around the flavin mononucleotide (FMN) chromophore to mimic the As^3+^ binding motif, and variants of these engineered light-oxygen-voltage (iLOV) proteins could sense As^3+^ with a relative high sensitivity (0.1 mM LOD). Recently, genetically encoded biosensors based on engineered FPs for the detection of As^3+^ have also been reported. Bokhari et al. developed two probes based on a bacterial As^3+^ responsive transcriptional factor AfArsR from *Acidithiobacillus ferrooxidans*. FRET-based biosensors were constructed by inserting AfArsR between FP acceptor/donor FRET pairs. Unfortunately, this method has not been applied to intracellular detection.

### 4.2. Aptamers

**Figure 7:**
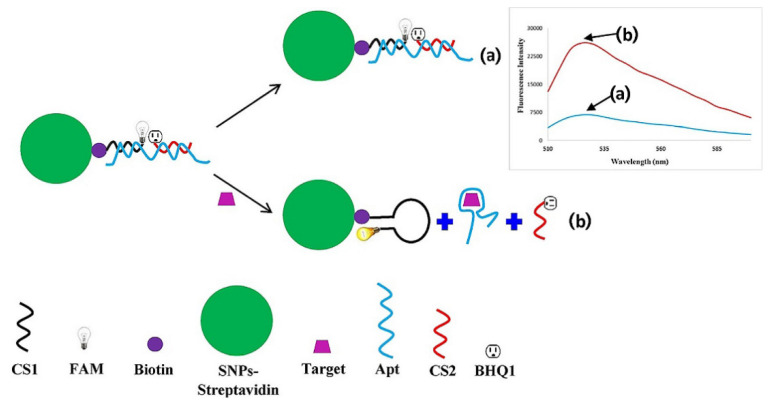
Function process of the developed fluorescent aptasensor for As (III) detection [80]. (a) In the absence of As (III), the structure of CS2-Apt-CS1 was complete and the distance between FAM and BHQ1 was narrowed, resulting in weak fluorescence intensity; (b) In the presence of As (III), Apt released its CSs and CS1 formed a stem-loop structure, resulting in a strong fluorescence intensity. Reproduced with permission from Ref. [80]. Copyright 2018 Elsevier.

Although some arsenic probes using aptamers have been introduced in the previous sections [33,41,51], the applicability of aptamers is emphasized here. Aptamers are small ssDNA or RNA molecules that are obtained via an in vitro process known as Systematic Evolution of ligands by Exponential enrichment (SELEX). As shown in Figure 7, Abnous et al. reported a fluorescent aptamer sensor that could be used for As^3+^ detection. The sensor consisted of silica nanoparticles coated with streptavidin (SNPs-streptavidin), FAM-labeled complementary strand of aptamer (CS1), unlabeled aptamer (Apt) and CS2 with quenching fluorophore (BHQ1) [80]. In the absence of As^3+^, Apt hybridized with CSs, and BHQ1 quenched the fluorescence to produce a weak fluorescence signal. After the addition of As^3+^, it bound to Apt to release CSs, and CS1 formed a hairpin structure on the surface of SNP chain avidin, leading to an enhanced fluorescence intensity. This was a typical fluorescent “on–off” sensor with an LOD of 0.45 nM.

In addition, due to the small number of biomolecules involved in the reaction and the limitations of detection methods, it is difficult to completely distinguish the resulting signal from the background signal. Therefore, it is necessary to use signal amplification technology to amplify the generated signal so that it can be well distinguished from the background signal. Here, a label-free fluorescence sensing platform was carefully designed to monitor As^3+^ using an exonuclease III (Exo III)-assisted cascade target recycling amplification strategy [81]. The sensor used a triple-helix molecular switch as the sensing element and 2-amino-5,6,7-trimethyl-1,8-naphthyridine as the signal indicator. The constructed sensor had high sensitivity and selectivity, with an LOD of 5 ng/L. Similarly, Chen et al. also reported a highly sensitive fluorescent biosensor with an Exo III-mediated amplification strategy for DNA cycling to detect As^3+^ [82]. In this sensor, the aptamer of As^3+^ was used as the recognition unit, and in the presence of As^3+^, the blocked DNA was released to trigger the cascade signal amplification process. DNA recycling in the presence of Exo III and catalytic cleavage based on DNAzyme resulted in the generation of a significantly amplified fluorescence signal for the highly sensitive quantification of trace As^3+^ with an LOD of 2 pM.

### 4.3. Bacteria

Bacterial biotransport technology is a new method to detect arsenic in water samples and food. For the detection of low concentrations of arsenic, arsenic-responsive bacterial organisms were reported to show far better performance than most chemical field test kits. The advantages of the technique are easiness of manipulation and preparation of (live) cells, high sensitivity of the cells, response well below 10 μg/L, and possible multiple reporter outputs. For example, there have been studies regarding biological reporters that produce fluorescence, bioluminescence, visible color, current, or pH changes [83,84,85,86].

A microfluidic chip containing an immobilized *Escherichia coli* (*E. coli*) biosensor that could express green fluorescent protein (GFP) under the exposure of arsenic was reported back in 2009 [87]. Similarly, Buffi et al. reported a biosensor based on genetically engineered bacteria (non-pathogenic laboratory *E. coli* strains) that produced variants of GFP upon exposure to arsenite and arsenate [88]. *E. coli* biotransporter cells encapsulated in agarose-bead and integrated into microfluidic devices could be used for the detection of arsenic in water samples. A portable biosensor containing a small polydimethylsiloxane microfluidic chip was reported to measure As^3+^ concentration in water using *E. coli* biotransport cells, with GFP expressed by *E. coli* as a signal [89]. The device had a complete optical illumination/collection/detection system for automatic quantitative fluorescence measurement. A whole-cell *E. coli* MG1655 biotransporter based on GFP was constructed to measure bioavailable arsenite and arsenate in water [90]. Sensor plasmid was constructed with a transcriptional fusion between the operator/promoter region of *ars* operon and *arsR* regulatory gene of *E. coli* plasmid R773 and the promoterless *gfpuv* (mutant form of GFP) reporter gene. Holmes et al. reported a whole-cell biosensor for the specific detection of bioavailable arsenic in E. coli by placing the GFP reporter gene under the control of the ArsR1 (GSU2952) regulatory circuit of *Geobacter Sulreducens* [91]. Under optimal test conditions, the LOD of arsenite detected by *Geobacter arsR1* promoter was 0.01 μM.

In addition to using *E. coli*, there are still some other types of bacterial biosensors. Huang developed a bacterial biosensor for the simultaneous detection of multiple bioavailable heavy metals (As^3+^ and Hg^2+^) [92]. The biosensor offered a choice of two reporting systems, *luxCDABE* and *gfp*, which were combined with metal response regulatory elements, respectively. The results showed that the induction of *LuxCDABE*-based constructs was more sensitive than that of *gfp*-based constructs in detecting As^3+^ and Hg^2+^. The sensor could distinguish between arsenic and mercury concentrations in groundwater samples to meet groundwater quality standards. Loprasert et al. developed a bacterial biosensor based on formylglycine-generating enzyme (FGE)-sulfatase with an integrated chromosomal sensing cassette for quantitative detection of arsenic in the environment [93]. The arsenic responsive promoter was obtained from the *Bacillus cereus* strain ATCC14579. The biosensor showed excellent performance in detecting low trace arsenite and arsenate.

## 5. Conclusions and Future Perspectives

In a word, the increasing toxic arsenic pollution seriously threatens the ecological environment and human health, so it is essential to accurately determine arsenic in the environment and organisms. Therefore, the design and synthesis of novel arsenic sensors with high sensitivity, high selectivity and good biocompatibility is still one of the hot topics in chemistry, biomedicine and other disciplines. In this review, we classified and reviewed the sensors for the determination of inorganic arsenite and arsenate by fluorescence spectrometry according to different principles. Among these sensors and biosensors, nanomaterials, organic molecules and biomolecules with unique optical properties were introduced to design and develop new sensing methods to obtain a significantly enhanced performance, including sensitivity, selectivity, cost, response time, practicality and convenience. It should be noted that the development of novelty sensors has led to the vigorous development of optical strategies for arsenic detection. Although the optical sensors for arsenic detection have been developed rapidly, there are still some problems that need further research and exploration. For example, these fluorescent probe strategies often rely on intelligent probe design and complex signal processing. Most of the reported sensors for detecting arsenic have only been utilized in the laboratory. It may be difficult to accurately monitor toxic arsenic in the real environment by applying these reported methods in view of the complexity of real samples.

According to the current research status, it is expected to further study and develop a new type of fluorescent arsenic sensor and explore its applications from the following aspects: (1) Strengthen the construction of a new fluorescence sensor to improve the stability and reliability of output signal. The sensor with multi-signal response and the pep-tide-based sensor with good biocompatibility have great potential. The microfluidics concept provides an excellent platform for the development of ideal arsenic detection sensors, integrating multiple technologies on a chip to enable a multi-signal response and support selective and ultra-sensitive arsenic detection. In addition, the peptide-based heavy metal ion sensor has become one of the effective methods to detect arsenic ions in the environment and living cells, as well as being applied in bioimaging, due to its advantages such as multiple coordination sites chelated with metal ions, variable amino acid sequences, good biocompatibility and cell penetration. (2) Improve the detection and anti-interference ability of actual samples. Unfortunately, most of the sensors in question interfered with metal ions or anions, which severely affects arsenic recognition. For sensors with strong anti-interference ability, the detection limit usually cannot meet the WHO guidelines. (3) Develop portable and functional sensors for arsenic detection based on chip technology to meet commercial requirements. The highest evaluation criterion of the sensor is whether it can be applied to the field detection or not, so how to advance the potential nanomaterial-based on-site analysis strategies for toxic arsenic is a big challenge. Fortunately, these strategies can be combined with emerging tools (3D printing, smartphone and handheld devices) to enable rapid field detection of arsenic. We believe that with the continuous exploration of arsenic detection sensors, the discovery of new strategies will further promote its development and applications.

## Figures and Tables

**Table 1:** Some fluorescence sensors based on nanomaterials for arsenic detection.

Detection System	Detection Mode	LOD	Ref.
Cys-Cys modified water-soluble fluorescent gold clusters	Turn-On	53.7 nM	[24]
Carbon-Au-BSA	Turn-Off	0.05 nM (0.004 ppb)	[25]
PLNPs and DTT modified AuNPs	Turn-On	55 nM	[26]
Multicolor fluorescent sulfur doped CDs	Turn-Off	0.032 nM (32 pM)	[27]
GSH-CDs	Turn-Off	2.3 nM	[28]
*O*-phenylenediamine and pyrazole CDs	Turn-Off	24.4 nM	[29]
Ag-h-CdS/ZnS	Turn-Off	3 nM (0.226 µg/L)	[30]
CdSe/QDs/Tb-GMP	Turn-On	5.2 nM (0.39 ppb)	[31]
MSA-CdTe/QDs	Turn-Off	214 nM (0.016 mg/L)	[32]
MPA-CdTe@CdS/QDs	Turn-Off	0.24677 nm (246.77 pM)	[33]
PbS/QDs	Turn-Off	49.4 nM (3.7 ppb)	[34]
ssDNA-CuInS_2_ QDs	Turn-On	0.13 nM	[35]
ssDNA-QDs	Turn-On	1.6 nM (0.2 ppb)	[36]
Fe-GQDs	Turn-On	68 nM (5.1 ppb)	[37]
Ce-CPNs	Turn-Off	9.3 nM (0.7 ppb)	[38]
FAM-labeled Ars-3 ssDNA	Turn-On	18 nM	[39]
Cd/Zr-UiO-66	Turn-Of	5400 nM (5.4 μM)	[40]

## Data Availability

Not applicable.
